# Inter-observer variability in contouring the penile bulb on CT images for prostate cancer treatment planning

**DOI:** 10.1186/1748-717X-6-123

**Published:** 2011-09-24

**Authors:** Lucia Perna, Cesare Cozzarini, Eleonora Maggiulli, Gianni Fellin, Tiziana Rancati, Riccardo Valdagni, Vittorio Vavassori, Sergio Villa, Claudio Fiorino

**Affiliations:** 1 Medical Physic, San Raffaele Scientific Insitute, Milano, Italy; 2 Radiotherapy, San Raffaele Scientific Insitute, Milano, Italy; 3 Radiotherapy, Ospedale Santa Chiara, Trento, Italy; 4 Prostate Program, Scientific Directorate, Fondazione IRCCS Istituto Nazionale dei Tumori, Milano, Italy; 5 Radiotherapy, Cliniche Gavazzeni Humanitas, Bergamo, Italy; 6 Radiotherapy, Fondazione IRCCS Istituto Nazionale dei Tumori, Milano, Italy

**Keywords:** Inter-observer variability, penile bulb, prostate cancer

## Abstract

Several investigations have recently suggested the existence of a correlation between the dose received by the penile bulb (PB) and the risk of erectile dysfunction (ED) after radical radiotherapy for clinically localized prostate carcinoma.

A prospective multi-Institute study (DUE-01) was implemented with the aim to assess the predictive parameters of ED. Previously, an evaluation of inter-observer variations of PB contouring was mandatory in order to quantify its impact on PB dose-volume parameters by means of a dummy run exercise.

Fifteen observers, from different Institutes, drew the PB on the planning CT images of ten patients; inter-observer variations were analysed in terms of PB volume variation and cranial/caudal limits. 3DCRT treatment plans were simulated to evaluate the impact of PB contouring inter-variability on dose-volume statistics parameters. For DVH analysis the values of PB mean dose and the volume of PB receiving more than 50 Gy and 70 Gy (V50 and V70, respectively) were considered. Systematic differences from the average values were assessed by the Wilcoxon test.

Seven observers systematically overestimated or underestimated the PB volume with deviations from the average volumes ranging between -48% and +34% (p < 0.05). The analysis of the cranial and caudal borders showed a prevalence of random over systematic deviations.

Inter-observer contouring variability strongly impacts on DVH parameters, although standard deviations of inter-patient differences were larger than inter-observer variations: 14.5 Gy versus 6.8 Gy for mean PB dose, 23.0% versus 11.0% and 16.8% versus 9.3% for V50 and V70 respectively.

In conclusion, despite the large inter-observer variation in contouring PB, a large multi-centric study may have the possibility to detect a possible correlation between PB % dose-volume parameters and ED. The impact of contouring uncertainty could be reduced by "a posteriori" contouring from a single observer or by introducing Magnetic Resonance Imaging (MRI) in the planning procedures and/or in improving the skill of observers through post-dummy run tutoring of those observers showing large systematic deviations from the mean.

## Background

Erectile dysfunction (ED) is known to be an adverse side-effect after radiotherapy for prostate cancer [1-14]. The growing fraction of young patients interested in conserving their potency is leading clinicians and researchers to devote more attention to this issue, as preservation of erectile functionality can have a significant impact on the quality of life of quite a large number of patients likely to be long survivors after curative radiotherapy for prostate cancer. From literature there is some evidence of a vascular ethiopathogenesis of radiation-induced ED, suggesting that irradiation of the penile bulb (PB), the crura and the corpora cavernosa could cause post radiotherapy ED [15-27].

Despite advances in treatment modalities, such as the use of intensity modulated radiation therapy (IMRT), leading to better sparing of the erectile structures involved in erection [28-31], the reduction of ED after radiotherapy remains a major concern in the Radiotherapy of prostate cancer.

Although it has been suggested that post radiotherapy ED may be related to the unnecessary irradiation of erectile structures, dose constraints have not yet clearly assessed, as recently reported in several reviews [32-34]. Possible clinical causes of the differences reported in a number of studies have been associated with the difficulty in assessing ED, the use of anti-impotence drugs and of hormonal therapy.

Moreover, due to the particular position of the erectile structures, mainly the penile bulb, technical/dosimetry uncertainties could play a role: firstly, the position of PB next to the caudal limit of the irradiation field may introduce additional uncertainty due to the day-by-day set-up position of the beams. Still more important could be the uncertainty in delineating PB and other erectile structures, especially with computed tomography (CT), owing to the recognised limits of this imaging technique, mainly the low contrast in the pelvic area. Due to these limitations, Magnetic Resonance Imaging (MRI) has been proposed as the most appropriate imaging modality for accurate localisation of the erectile structures [31,35-44].

In a previous study, Perna et al. [31] evaluated the impact of various imaging modalities and treatment techniques for prostate tumours. The Authors demonstrated that MRI is superior to CT with regard to soft tissue contrast. This technique was consequently found to lead to better sparing of PB mainly due to a more precise definition of the prostate apex.

Nevertheless, CT scan is still widely used for prostate cancer planning, being the standard imaging technique for most radiotherapy Institutes where access to MRI for planning is still lacking. Although the well documented limitation of CT images in the delineation of the penile bulb could impact on the PB dose volume parameters, no specific studies have been conducted to date on this important issue.

In April 2010 a prospective multi-Institute study (*Disfunzione Urinaria Erettile*, DUE-01) was activated after approval from the ethics committee. The purpose of the DUE-01 study is to prospectively assess the predictive parameters of genito-urinary toxicity and ED, including the possible correlation between ED and PB dose-volume parameters. In this type of study, involving many Institutes aiming at evaluating the possible correlation between normal tissue complication and dose distribution, it is mandatory to investigate the impact of contouring uncertainties on dose-volume parameters. Accordingly, the first step of the DUE-01 study was the activation of a dummy run exercise for the contouring of PB with the aim of: 1) assessing the impact of contouring uncertainty on PB dose-volume parameters potentially predictive of ED; 2) suggesting possible methods/strategies to minimise their impact; 3) giving individual recommendations to reduce inter-observer variations in case of operators "significantly far from the average".

## Methods

CT images of ten prostate patients were randomly selected for the dummy run exercise. Axial CT images of the pelvis were acquired with a General Electric System using 110-130 kV and 200-250 mA, 4 mm slice thickness and 512 × 512 matrix, extending from the level of the sacrum to below the ischiatic tuberosities. CT scans were performed on patients with a full urinary bladder and an empty rectum without any contrast medium. Patients were placed in the supine position on a flat couch; legs were slightly flexed with feet immobilized in a foot support combi-fix (Civco Orange City IA, USA).

Fifteen physicians involved in the treatment of prostate cancer in the different Institutes enrolled in the DUE-01 study were asked to draw the PB.

**Figure 1:**
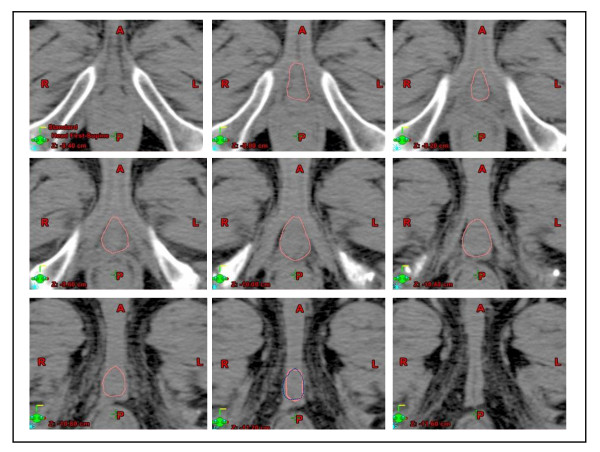
**Example of the contouring of the penile bulb, based on the guidelines suggested by the steering committee**. The projection of the PB contour on the more caudal slice from the PB drawn on the more cranial slice is shown by a blue dashed line. This tool was used to copy the anterior border of the PB contour from the more cranial slice onto the more caudal slice.

Before starting the dummy run, both patients and observers were anonymised. In order to standardise the PB definition, all physicians were instructed to adhere strictly to the following guidelines previously defined by the steering committee of the study: apart from the definition of well-known anatomic boundaries (the paired crura laterally, the corpora spongiosum anteriorly and the levator ani posteriorly) [45], because of the low contrast on CT images in the pelvis area, the anterior border of PB in the more caudal slices was arbitrarily defined as the projection of the PB anterior border of the most caudal slice, where this border is more visible. In order to facilitate the observers, one sample patient with his PB drawn following these guidelines (see Figure 1) was shown before the contouring session.

PB contours were drawn using the treatment planning system (TPS Eclipse-Aria, Varian Inc.) installed at San Raffaele Hospital - Milan, the coordinating Centre of the study.

During contouring, every operator was blinded to the others and optionally used TPS tools such as zoom, window/contrast level and copying contours, showing contours on adjacent slices and projected contours on saggital/coronal view reconstruction.

In order to evaluate the impact of contouring inter-variability on dose-volume histogram (DVH) parameters, for each patient, a prostate treatment plan was simulated. The plan simulations were performed using an 18 MV X-Ray four field box technique, prescribing a dose of 76 Gy to the original PTV. Dose distributions were calculated using the pencil beam model implemented in the TPS, with modified Batho inhomogeneity correction. Grid size used for calculation was 2.5 × 2.5 mm.

PB inter-observer variations were analysed in terms of volume differences and cranial/caudal limit variations.

For DVH analysis the values of PB mean dose and the volume of PB receiving more than 50 Gy and 70 Gy (V50 and V70, respectively) were collected for each patient and each observer, both as absolute (cc) as well as relative (%) values. The rationale for the selection of these DVH parameters was that mean dose and V(50) as surrogate of a threshold dose for ED, whereas V(70) was representative of the overlap between the penile bulb and the target volume.

For each parameter considered, the average difference between observer and mean values were tested with a non-parametric Wilcoxon matched-pairs test; p-values lower than 0.05 were considered statistically significant. All statistical analyses were performed using SPSS v.17 software.

Finally, the standard deviation of the differences between each observer and the average value was calculated for each patient *j *(SD_IO,J_) for all considered dose-volume parameters (%V50, %V70; ccV50; ccV70; Mean PB dose): the global inter-observer variability (SD_IO_) was assessed for each parameter as (Σ_J_SD_IO,J_)/N, where N is the number of considered patients (i.e.: N = 10).

For comparison, inter-patient variability was assessed for each parameter as the SD of the mean value over the ten considered patients (SD_IP_), taking the mean values of all observers for each patient.

## Results

**Figure 2:**
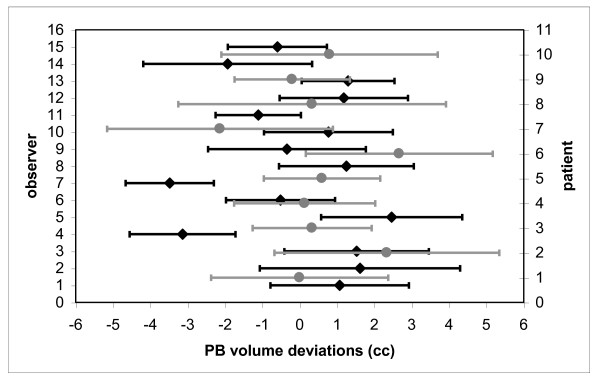
**Average deviations and SD between each observer (black) and each patient (grey), and the average value for PB volume**.

Figure 2 shows the mean values and standard deviations (SD) of the differences between each observer and the average value of PB volume. In this figure the mean values and standard deviation of the differences of PB contour for each patient are also reported.

Seven observers [3-5,7,11,13,14] overestimated or underestimated PB volume with significant deviations (p < 0.05) from the average volumes ranging between -5 cc and +4 cc. Due to the small volume of PB (around 5 - 20 cc) these differences emphasise a great inter-observers variation (-48% and +34%). In particular, observer 5 overestimated PB volume for all patients while, on the contrary, observers 4 and 7 had a tendency to grossly underestimate PB volume.

These high deviations could be explained by a lower quality of CT images for some patients. An analysis of inter-observers variability patient-based showed that patients 2, 6 and 7 are "worse".

**Figure 3:**
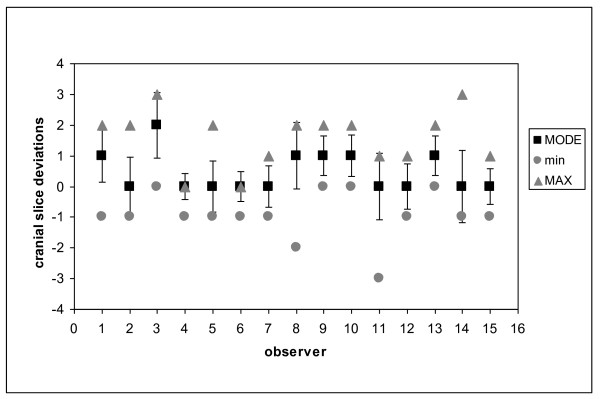
**Deviations between each observer and the most probable value for the cranial slice of PB**.

**Figure 4:**
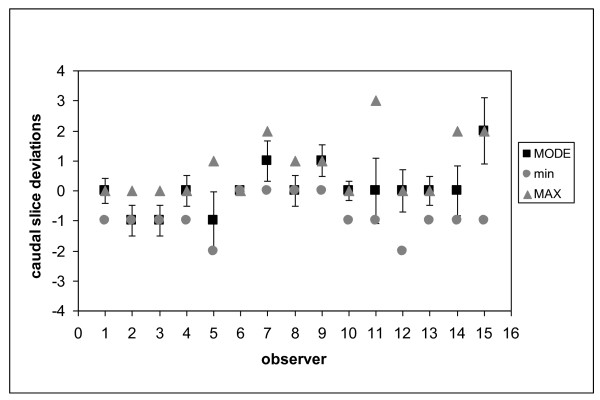
**Deviations between each observer and the most probable value for the caudal slice of PB**.

Differences in cranial and caudal limits of PB contours are shown in figures 3 and 4 respectively. The deviations were expressed in terms of the average discrepancy and SD from the slice most frequently drawn from each observer. Maximum values (the more cranial slice) and minimum values (the most caudal slice) are also indicated in the figures. Regarding the cranial limit, four observers [3,9,10,13] showed a systematic (p < 0.05) deviation.

As concerns the caudal border, significant deviations were found for observers 2 and 3, whose contouring was approximately 1 slice more caudal, whereas observers 9 and 15 defined the caudal border more cranially by, on average, 1 and 2 slices respectively.

An analysis patient-based of the cranial and caudal borders, although detected some random variations, did not show significant systematic differences; therefore, presumably, most deviations were in the lateral and/or anterior-posterior directions

**Figure 5:**
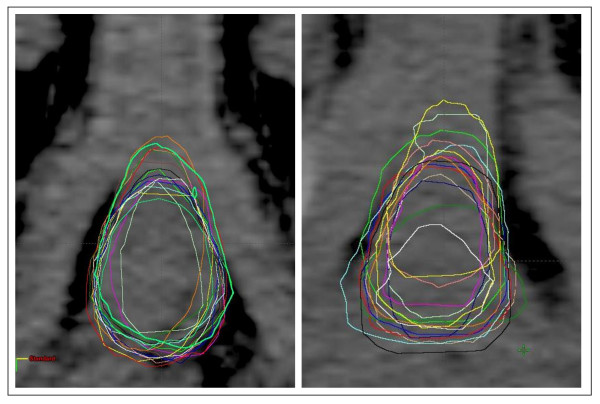
**A plot of the central slice of PB contours drawn by all observers of two patients: one with the lowest inter-observer volume variation (left side) and one with the largest inter-observer volume variation (right side)**.

A plot of the central slice of PB of two patients (one with the lowest and one with the largest inter-observer volume variation) is shown in Figure 5.

The impact of inter-observer variations on dose statistic and DVH parameters was great. The differences in mean dose of PB among the observers ranged mostly from -20% to +20%. The differences from mean values were statistically significant for observers 2, 3, 4, 6, 9 and 12.

A similar trend was evident for DVH parameters: the differences concerning V50 ranged from -11% to +9% (-2 cc - +2 cc) with p-value statistically significant for 10 out of 15 observers, whereas, concerning V70, the statistically significant differences were, for 5 out of 15 observers [3,4,6,7,12], in the range -8% - +8% (-1 cc - +1 cc).

**Figure 6:**
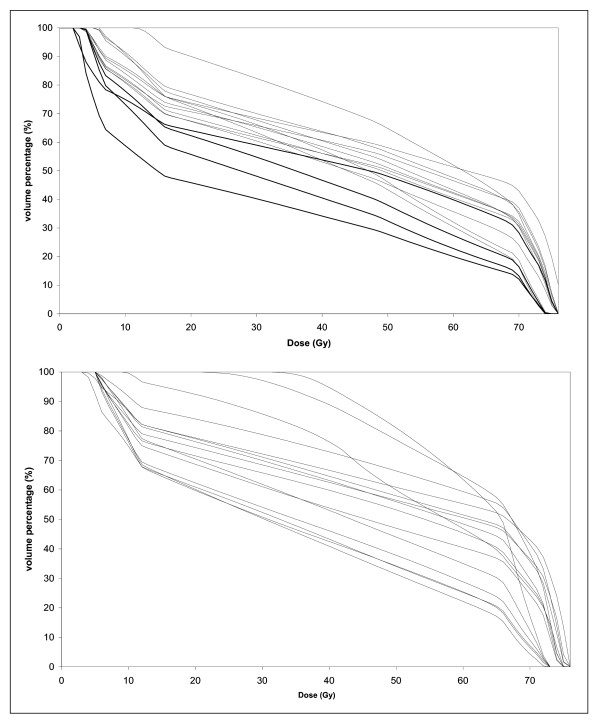
**Graphs showing PB dose-volume histograms relative to the two patients in figure 5: the first (top of figure) with the lowest impact of inter-observer variation on DVH parameters, and the second (bottom of the figure) with the largest impact of inter-observer variation on DVH parameters**.

Figure 6 shows the graphs of PB dose-volume histograms relative to the two patients in figure 5: the first with the lowest impact of inter-observer variation on DVH parameters and the second with the greatest impact of inter-observer variation on DVH parameters.

**Figure 7:**
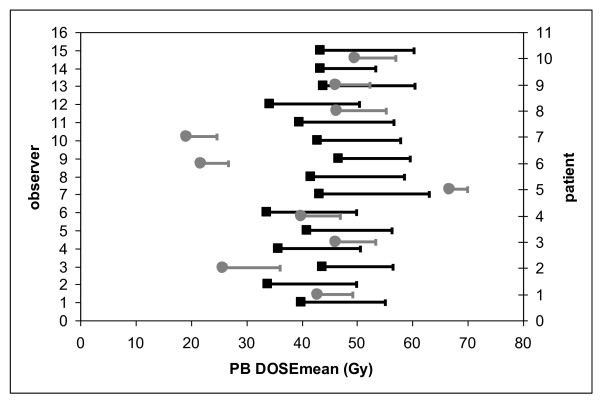
**Average value and standard deviation of PB dose mean (Gy) for each observer (black) and each patient (grey)**.

**Figure 8:**
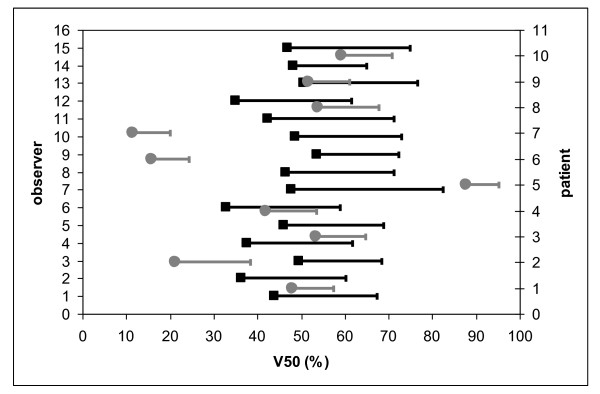
**Average value and standard deviation of V50 (%) for each observer (black) and each patient (grey)**.

**Figure 9:**
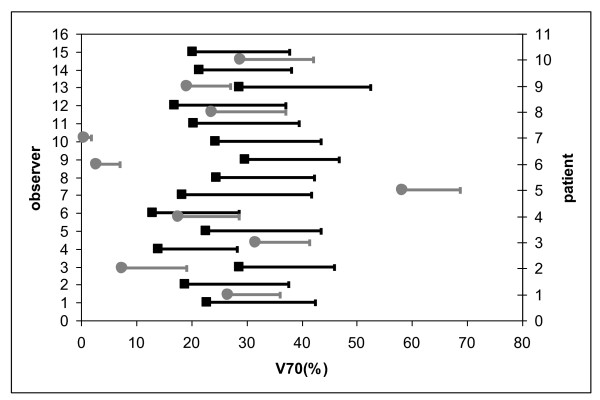
**Average value and standard deviation of V70 (%) for each observer (black) and each patient (grey)**.

When considering %DVH parameters, inter-patient differences were larger than inter-observer differences (see Figures 7, 8 and 9). SD_IP _and SD_IO _were respectively 14.5 Gy and 6.8 Gy for mean PB dose; 23.0% and 11.0% for V50; 16.8% and 9.3% for V70.

On the contrary, when considering absolute (cc) DVH parameters, the impact of inter-observer variability was comparable with inter-patient variability: 1.38 cc versus 1.41 cc for V50 and 1.00 cc versus 1.03 cc for V70.

No correlation could be demonstrated between %/cc DVH variations and PB volume/limit variations.

## Discussion

The contouring uncertainty in dose-volume modelling studies has been generally neglected, or at least under-reported/under-estimated, despite its potentially impact.

An important general point concerns the need for clear and simple guidelines for organ delineation; a successful application of such guidelines was demonstrated in the case of the rectum in rectal toxicity dose-volume relationship studies where a robust anatomically based definition of the cranial and caudal borders of the rectum guaranteed sufficient reliability of the DVHs collected in a large multi-centric trial [46-48].

The potential impact of PB contouring uncertainty in the context of prostate cancer radiotherapy presents a number of special features: in particular, the proximity of PB to the caudal limit of the PTV suggests that PB dose-volume parameters are highly sensitive to this uncertainty. Another specific point concerns the fact that slight deviations in contouring among different observers lead to large relative changes in volume and DVH parameters, due to the relatively small volume (generally between 5 and 20 cc) of this structure.

The quantification of PB contouring uncertainty is of primary importance in multi-centric studies dealing with dose-volume parameters and ED; surprisingly, no data are available on this point.

The recent start-up of the prospective DUE-01 study represented a good opportunity to investigate this issue for the first time; despite the superiority of MRI to CT in defining PB, it was decided to assess inter-observer variation in contouring PB on CT images, as this technique still represents the routine practice for most institutes in Europe, although the use of MRI for prostate planning is increasingly widespread.

The main result of the current investigation is the quite large contouring variability, even in the presence of a much discussed and well-accepted protocol for PB drawing, including a simplified definition of the anterior border. Depending on the visibility of PB, which varies from patient to patient, great uncertainty in terms of volume variations could be seen. On the other hand, although all the observers were highly skilled in prostate radiotherapy planning, many do not routinely contour PB in their own Institute; this could partially explain the high level of uncertainty.

The practice of contouring PB and/or other penile structures has yet to be consolidated, as only in recent years has the problem of ED after radiotherapy for prostate cancer been truly addressed.

There is evidence that the dose received by PB could be predictive of ED, but the literature reports a number of controversial results [23-27]; on the other hand, the Roach et al. paper [24] and other results have been very important in recent years in focusing on the possible clinical advantage deriving from the sparing of PB and other erectile structures.

It is quite likely that the increasingly common practice of contouring PB as an organ-at-risk for potent patients will rapidly lead to a significant reduction of contouring variability, such that our results should be considered as a photograph of the present situation. The increased use of MRI, too, will likely help in reducing contouring uncertainty, as demonstrated by evidence that this imaging modality is highly superior to CT in defining PB and other penile structures.

As a consequence of volume variability, the impact of contouring uncertainty on dose-volume parameters of PB was found to be great as well. An important result was that, without any intervention to reduce it, inter-observer variability of absolute (cc) DVH parameters is as large as inter-patient variability. This result shows that the dose-volume relationship for PB would be completely hidden only due to contouring uncertainty. On the other hand, inter-patient variability was found to be twofold larger than inter-observer variability when considering mean PB dose and % DVH parameters. As an example of the impact of inter-observer variability, with regard to the constraint V50 < 50%, our results (1 SD for inter-observer variability on V50: 11%) suggest that with a V50 value of around 35% there is still a probability of about 10% that V50 is higher than the constraint; inversely, if V50 is around 65%, there is a probability of about 10% that V50 is below our constraint. Although our result suggests a significant impact of contouring variability, in the presence of a large cohort of patients, as in the DUE-01 study, in which more than 500 patients are expected to be enrolled, the existence of a dose-volume relationship could be detected. Further investigation on the expected impact of these uncertainties on the predictive power of our study is warranted; in any case, it is clear that % DVH should be used to search for correlation, while absolute DVH should be ignored.

Attempts to reduce the impact of contouring variability are now in progress and include both a re-contouring after an MRI tutorial and specific advice to those observers for whom the largest systematic deviations from the average values of PB mean dose and % DVH were detected.

Another possible solution under discussion is the "a posteriori" contouring by a single observer, as planning CT information will be collected in the coordinating centre and analyzed with dedicated research software (Vodca, Inc).

## Conclusion

The dummy run showed very high inter-observer variation with significant differences in PB contouring among the various observers, also affecting dose-volume parameters and consequently the possible relationship with ED. The high variability should be possibly due to both the limitations of CT images (i.e. the low contrast of the soft tissues in the pelvis area) and the differing experience among observers in contouring the erectile structures. The very large impact on DVH mainly depends on the small PB volume and its critical position near the caudal border of the PTV. This study suggests that the reliability of the quantification of dose-volume effects of penile bulb defined on CT images may be significantly reduced in multi-institutional studies. Possible solutions may be the "a posteriori" contouring by a single observer, the introduction of MRI and/or improving the agreement among observers after critical review and repetition of the dummy run procedure.

## Competing interests

The authors declare that they have no competing interests.

## Authors' contributions

LP conducted the study, collected and analyzed data and wrote most of the manuscript; CC, SV, TR, RV, CF made important contributions in the design of the study and in revising the content; EM contributed in collecting and analyzing data; CF wrote parts of the manuscript and contributed in analyzing data.

All authors read and approved the final manuscript.
